# Differences in educational attainment between obese and non-obese Kuwaiti female university students

**DOI:** 10.1017/jns.2020.24

**Published:** 2020-07-24

**Authors:** Hanouf H. Al Hammadi, Hamad A. Alaslawi, Allan Hewitt, John J. Reilly

**Affiliations:** 1 School of Psychological Sciences and Health, University of Strathclyde, Glasgow G1 1XQ, UK; 2 Department of Sociology and Social Work, College of Social Sciences, Kuwait University, Kuwait

**Keywords:** Obesity, Adolescents, Body mass index, Kuwait, Academic achievement, BMI, body mass indexGPA, Grade Point Average

## Abstract

Individuals with obesity tend to perform less well than their non-obese peers in tertiary education, but there is little evidence from non-Western countries and recent studies. The present study aimed to test whether academic attainment differed between female undergraduates with obesity (defined by body mass index (BMI)), and those who were non-obese in Kuwait, a country with very high obesity prevalence. In 400 female Kuwaiti first- and second-year Social Science students (mean age 18⋅0, sd 0⋅6 years), educational attainment was defined as the Grade Point Average (GPA) across all subjects (from 1⋅00 to 4⋅00). The mean GPA (2⋅51, sd 0⋅53) among students defined as obese by the BMI (*n* 163) was significantly lower than among the students defined as non-obese by the BMI (*n* 237; 2⋅80, sd 0⋅63; *P* < 0⋅001), and those defined as obese were more likely to be in the lowest quartile for the GPA (OR 3⋅03; 95% CI 1⋅90, 4⋅85), independent of socio-economic status. Similar differences were observed between students defined as having high versus normal body fatness. Female undergraduates in Kuwait with obesity have lower academic attainment than their non-obese peers, and universities should consider measures to mitigate reduced attainment among their female undergraduates.

In Kuwait, more than 40% of adults have obesity as defined by the BMI^(1–5)^. The prevalence of obesity is also extremely high among Kuwaiti children and adolescents^(6,7)^. Obesity rates are still rising among Kuwaiti adults and children, costing the country a minimum of 2⋅8 billion dollars annually in both direct and indirect costs^(8)^; with a Kuwaiti population of only 1⋅1 million,^(9)^ Kuwait has about 5000 bariatric procedures each year^(10)^.

Obesity in childhood, adolescence and adulthood increases the risk of a large number of medical problems^(11–13)^. If obesity impairs educational attainment, this could provide new arguments for obesity prevention and treatment^(14–17)^. In the UK, for example, a large cohort study by Booth *et al.*^(14)^ found that obesity in adolescence was associated with markedly poorer academic attainment, independent of confounders, but in girls only. Recent systematic reviews have disagreed on the quality, quantity and consistency of the evidence on differences in academic attainment between individuals with obesity and those who do not have obesity: Santana *et al.* concluded that deficits in academic attainment in individuals with obesity might be explained largely by confounding (with obesity much more prevalent in individuals with lower socio-economic status in high-income Western countries) and called for more research on the issue^(15)^. Hill *et al.*^(16)^ concluded that deficits in academic attainment in undergraduates with obesity were fairly well established, particularly in women, and might be explained largely by weight-related bias. The Cochrane review by Martin *et al.*^(17)^ highlighted plausible mechanisms relating obesity to lower academic attainment including social mechanisms such as stigma/bias and impaired quality of life associated with obesity, biological mechanisms (such as cognitive effects of inflammation)^(18,19)^ and mechanisms related to poor health associated with obesity (such as illness-related absenteeism). An additional systematic review^(20)^ concluded that impaired academic attainment associated with obesity was most likely in adolescent girls and young adult women^(20)^.

Since systematic reviews on the topic have not reached consistent conclusions, other than the likelihood that obesity-related deficits in attainment may be worse in females than males, and almost all research included in those reviews comes from older studies in high-income Western nations, there is a need for new research from a wider range of settings. The primary aim of the present study was therefore to test whether educational attainment in Kuwaiti undergraduates was lower in individuals with obesity. We studied female undergraduates because of the previous evidence that obesity-related deficits in attainment might be worse in females than males.

## Methods

The present study recruited a convenience sample of first- and second-year Kuwaiti University College of Social Science students between March and May 2019. Sampling and recruitment have been described in detail elsewhere^(21)^. In brief, all first- and second-year Social Science undergraduates were invited to take part by the researcher. Students were considered suitable for inclusion if they were female, Kuwaiti nationals, <20⋅0 years of age and did not have any condition or illness which would have altered their weight status (e.g. pregnancy and long bone fracture). Students were excluded if they were male, non-Kuwaiti nationals, 20⋅0 years or older and had any condition or illness affecting their weight status, or reported any other chronic disease. The aim of these inclusion and exclusion criteria was to provide a relatively homogenous sample, and one which was relatively free of a number of potential confounders (e.g. non-Kuwaiti nationality and age). Of the 2169 students contacted, 525 expressed an interest and 400 of these were eligible and were entered into the study. All participants gave informed written consent to participation, and the study was approved by the University of Strathclyde Psychological and Health Sciences Ethics Committee.

### Measures of exposure (BMI and body fatness)

Anthropometric measurements and BMI calculations were carried out as described by Al-Hammadi and Reilly^(21)^. A Seca Stadiometer (Seca, London, UK) was used to measure the height to the nearest 0⋅1 cm. Weight was measured to 0⋅1 kg with study participants in light indoor clothing by the Tanita model TBF-310 (2625 South Clearbrook Drive Arlington Heights, IL, USA). A BMI *z*-score of ≥2⋅0 relative to WHO 2007^(22)^ reference data was used to define obesity in the adolescents (17–19⋅0) years of age (*n* 275) and for those over 19⋅0 years old the adult cut-off point of BMI ≥30 kg m^2^ was used (*n* 125)^(23)^.

### Educational attainment measurement

The measure of educational attainment outcome used in the present study was the Grade Point Average (GPA) for all university subjects. The GPA was provided in an anonymised form from the university authorities. The GPA was used in two ways: as a continuous variable (from 1⋅00 to 4⋅00) and a categorical variable, with the GPA distribution divided into quartiles from highest (Q1) to lowest (Q4).

### Potential confounders

In high-income Western countries, socio-economic status is a potential confounder of obesity–educational attainment relationships because it is associated with both higher risk of obesity and lower educational attainment^(15,20)^. Socio-economic status was considered as a potential confounder in the present study using parental educational attainment (degree education versus education to the high school level). Despite the relatively narrow age range, we also considered student age as a potential confounder.

### Statistical analysis

Data were analysed with SPSS version 26 (IBM Corp., Armonk, NY, USA) and Medcalc (Belgium). The data were tested for normality, and summary data were described as mean (standard deviation (sd)) or median (range) depending on the distribution of variables which was assessed by plots of the data and D'Agostino–Pearson tests in MedCalc. We compared the GPA between students categorised as obese by the BMI versus those considered non-obese by the BMI using two-sample *t*-tests. We also compared the percentage with obesity among the GPA quartiles using both *χ*^2^ tests and calculated odds ratios for risk of being in the lowest GPA quartile by obesity. *P*-values of <0⋅05 were used to indicate statistical significance.

The power of the present study was difficult to assess at the outset, and the power was fixed as this was part of a wider study of the ability of BMI to identify excessive fatness among female Kuwaiti adolescents^(21)^.

## Results

### Characteristics of study participants

A total of 525 students agreed to take part in the study, and 125 were excluded as they did not meet the inclusion criteria (age over 20⋅0 years, pregnancy, long bone fracture in cast and presence of chronic diseases). Therefore, 400 actually took part, and all 400 provided data for all variables. Of 400 participants, 163 were defined as obese based on the BMI and 247 were defined as excessively fat based on the bio-impedance measure.

### Educational attainment differences between individuals with obesity and those who did not have obesity

The mean GPA in the overall sample of 400 was 2⋅68 (sd 0⋅62). The mean GPA in the sample with obesity defined by the BMI (*n* 163) was 2⋅51 (sd 0⋅53; 95% CI 2⋅42–2⋅59), and the mean GPA in participants defined as not having obesity according to their BMI (*n* 237) was 2⋅80 (sd 0⋅65; 95% CI 2⋅71, 2⋅88). This difference was statistically significant (*P* < 0⋅001; 95% CI for difference in means = 0⋅17, 0⋅41).

A *χ*^2^ test on the distribution of GPA quartiles by obesity versus non-obesity status using the BMI was statistically significant (*P* < 0⋅001; Table 1). The odds ratio, unadjusted, for risk of being in the lowest quartile of GPA in the individuals with obesity according to the BMI was 3⋅03 (95% CI 1⋅90, 4⋅85; *P* < 0⋅001). Student age and parental educational attainment were not associated with the exposure and outcome values, and did not confound the relationship between BMI-defined obesity and the GPA.

**Table 1.** Grade point average (GPA) quartiles by weight status

	Quartile 1 Highest	Quartile 2	Quartile 3	Quartile 4 Lowest
GPA range	4⋅00–3⋅00	3⋅00–2⋅70	2⋅70–2⋅10	2⋅10–1⋅00
Mean GPA (sd)	3⋅44 (0⋅32)	2⋅92 (0⋅11)	2⋅48 (0⋅16)	1⋅88 (0⋅27)
Obese by the BMI *n* 163	28	35	39	61
Non-obese *n* 237	72	65	61	39

## Discussion

### Main findings and study implications

The present study found that undergraduates with BMI-defined obesity had poorer overall academic attainment than those who did not have obesity, and this difference could not be explained by socio-economic status. The impact of obesity on educational attainment might be helpful in both obesity prevention and treatment^(14)^. Individuals and families may be motivated to change weight status or health behaviours for cognitive or educational benefits. The very high prevalence of obesity in Kuwait, combined with the importance of educational attainment, might therefore provide new/additional arguments for obesity prevention and treatment in Kuwait and the other Gulf States. Universities should also be more aware of the increased risk of poorer attainment among undergraduates with obesity and have a particular responsibility to do so if at least some of this poorer attainment relates to bias or stigmatisation from peers and/or university staff^(16)^. The magnitude of the difference in the GPA between groups in the present study might also be sufficient to motivate universities to address the issue in future, even if only to raise student attainment.

### Comparisons with other studies

Recent systematic reviews^(15–17,20)^ have generally concluded that obesity pre-disposes to lower academic attainment, particularly in girls and women. However, some of these reviews have questioned whether this might be explained by confounding by socio-economic status, with poverty being associated with both lower academic attainment and obesity in high-income Western countries. Systematic reviews and original studies have identified a number of plausible mechanisms by which obesity might impair educational attainment. Potential mechanisms^(15–17,20,24–27)^ include increased absenteeism from university or school associated with the co-morbidities of obesity; cognitive deficits associated with cardiometabolic co-morbidities; impaired quality of life and the psychosocial co-morbidities of obesity; the impact of obesity on brain structure and/or function particularly in the pre-frontal cortex and hippocampus; the impact of obesity on behaviours known to be associated with educational attainment such as reduced moderate–vigorous intensity physical activity or less healthy diet and weight stigmatisation by peers or teachers. The present study was designed to test whether academic attainment was lower in female undergraduates with obesity and was not designed to identify mechanisms underlying this difference. However, some cultural and socio-economic differences between Kuwait and high-income Western countries might help in the development of future research aimed at understanding why the differences observed in the present study exist. First, poverty is almost non-existent among Kuwaiti nationals, and income inequality is extremely low in Kuwait compared with high-income Western countries^(28,29)^. There is also some evidence that obesity is not confounded by socio-economic status in Kuwait, in contrast to Western countries^(30)^. Weight stigmatisation and psychosocial impacts of obesity among adolescent girls and adult women may also differ between Kuwait and other nations. There is limited evidence on such differences to date, but impaired quality of life is the norm among adolescents with obesity in Western countries, but not present in Kuwaiti adolescents^(31)^.

### Study strengths and weaknesses

The present study had a number of strengths. First, the focus on adolescent girls and women, the groups most likely to experience obesity-related impairment of educational attainment, was important. Second, the novelty of the study was high because of the non-Western setting and the contemporary sample (with most of the previous literature from undergraduates from an era when obesity prevalence and access to tertiary education were very different^(16)^). Third, we examined potential confounding by socio-economic status, considered crucial by a number of previous reviews, notably Santana *et al.*^(15)^

The present study used the BMI to define obesity (obesity is a high level of body fatness rather than a high BMI)^(21)^ – while the BMI is a convenient proxy for high body fatness, it has only moderate sensitivity for high body fatness in Kuwait and in other populations^(21)^. In an attempt to address this potential weakness, we also tested for differences in academic attainment between individuals defined as having high body fatness (≥30% of body weight, as estimated by bio-electrical impedance) versus those with lower body fatness in the present study. This analysis is summarised in Supplementary Table S1 of Supplementary material, but results and conclusions were very similar to those from the analysis based on BMI-defined obesity. The power of the study was difficult to assess at the outset, but the sample size was sufficient to detect significant associations. The present study sample of 400 undergraduates was derived from a potentially eligible population of about 1800, and the extent to which biases in recruitment to the study affected study findings are unclear. Defining socio-economic status by income is problematic in Kuwait with such limited variation in income relative to Western countries^(28–30)^ – the present study used parental educational attainment as a convenient and relevant individual-level indicator of socio-economic status, but there is probably no ideal single measure. The present study was not designed to identify the mechanisms of any associations, and further studies will be required to do so. The generalisability of the findings is also unclear and will need to be tested in other studies. Finally, the present study had a cross-sectional design and so is restricted to identifying differences in academic attainment between individuals with obesity versus those who did not have obesity, and cannot confirm that obesity is a cause of lower academic attainment. However, the present study findings were consistent with a good deal of other evidence, there are plausible biological and social causal mechanisms, and reverse causality (lower educational attainment causing obesity and excessive fatness) is possibly unlikely in this case.

#### Conclusions

This research suggests that having obesity may impair academic attainment in Kuwaiti female students. Further studies will be needed to test the generalisability of these findings and to identify the underlying mechanisms of any effect of obesity on educational attainment.

## Supplementary material

For supplementary material accompanying this paper visit http://dx.doi.org/10.1017/jns.2020.24. click here to view supplementary material
