# Supplementary material for: Differences in educational attainment between obese and non-obese Kuwaiti female university students
Source: J Nutr Sci. 2020 Jul 24;9:e30. doi: 10.1017/jns.2020.24 (PMC7443771; doi:10.1017/jns.2020.24)
Supplement: Supplementary file 1 [file S2048679020000245sup001.docx]

**Supplementary Table 1.**

**Differences in Educational Attainment (Grade Point Average, GPA) Between Individuals With High Body Fatness vs Those With Lower Body Fatness**

High body fatness was defined as a body fat % of >30.0 derived from bio-electrical impedance^21^.

Mean GPA in the sample with high body fatness (n= 247) was 2.59 (SD 0.59; 95% CI 2.52-2.67) and in participants defined as not having high body fatness (n=153) was 2.81 (SD 0.64; 95% CI 2.70-2.91). This difference was statistically significant (P<0.001; 95% CI for difference in means = 0.08 to 0.33).

A chi-squared test on the distribution of GPA quartiles by high fatness versus non-high fatness was statistically significant (P<0**.**001)**.** The odds ratio, unadjusted, for risk of being in the lowest quartile of GPA in the individuals with high fatness according, was 2.39 (95% CI 1.43-3.99 ; P<0.001). Student age and parental educational attainment were not associated with the exposure and outcome values, and did not counfound the relationship between body fatness and GPA.
